# Commentary: Understanding ESL Teachers' Agency in Their Early Years of Professional Development: A Three-Layered Triadic Reciprocity Framework

**DOI:** 10.3389/fpsyg.2022.890098

**Published:** 2022-04-19

**Authors:** Xiaohong Hu, Xinmin Zheng

**Affiliations:** ^1^School of English Studies, Shanghai International Studies University, Shanghai, China; ^2^School of Education, Shanghai International Studies University, Shanghai, China

**Keywords:** teacher agency, teacher development, teacher identity, teacher education, Hong Kong

We read with interest the recently-published article Huang and Yip (2021) that has provided a better understanding of English as a Second Language (ESL) teachers' agency in their early years of professional development, which ended up by proposing a three-layered Triadic Reciprocity Framework (based on Jenkins, 2020). As we see it, it is worth noting that the newly-proposed framework has demystified and specified teacher agency, especially by presenting different degrees of it, namely proactive agency, reactive agency, and passive agency. Also, the article has successfully raised concerns over the sustenance of teachers' professional development. The article strongly suggests that favorable environment should be provided by school leaders and policy makers in order to help teachers enact proactive agency and then benefit their professional development. In light of its theoretical and practical values, we consider this article worthy of being commented on and shared to larger reader groups.

We believe that this study not only makes contributions to both research and theory, but also sheds light on how to diversify data sources when conducting qualitative research. Our reasons are stated as follows.

By reviewing the literature on language teacher agency, Tao and Gao (2021) underline that research applying the social cognitive theorization of agency to teacher agency remains limited and normally a quantitative design is adopted. However, since the dynamic (Imants and Van der Wal, 2020) and contextualized (Lasky, 2005) nature of teacher agency can't be ignored, a sociocultural approach is much needed for understanding the complex change process of teacher agency. Compared with previous research studies, this study examines the enactment and development of teacher agency by employing the Triadic Reciprocity Framework Core Agency Concepts (TRFCAC) model (Jenkins, 2020), along with a set of qualitative research methods to collect data, which stands out uniquely for its investigation of teacher agency from a combination of two theoretical perspectives, that's it, social cognitive theory and sociocultural theory.

As for theoretical contribution, this study has made improvements to the borrowed analytical framework by delineating the developmental nature of teacher agency and thus proposing a three-layered TRFCAC model. As mentioned previously, teacher agency is dynamic and context-specific. In other words, teachers may develop different degrees of agency, largely dependent on the concrete and specific contexts they involve themselves in. The newly-added layer to the adopted analytical framework has revealed the fact that teachers may undergo changes among proactive agency, reactive agency, and passive agency, which highly hinges on whether they are afforded or constrained by the contexts. We can therefore say that this newly-proposed framework enables us to understand teacher agency more properly and comprehensively.

Qualitative in nature, this study is well-triangulated to maintain its reliability and credibility. The main data collection activity was in-depth interviews, but this was complemented by personal communication and school documents. It is obvious that this method helped the researchers to “get a much more rounded view of the phenomena they are interested in” (Groom and Littlemore, 2011, p. 79). Thus, it turns out that the authors' interpretation of their understanding of teacher agency can be enhanced by peeking into the internal world of teachers through interviews and communication, supplemented by external materials like school documents.

Based on the analysis above, it is obvious that the current study has broadened the previous theoretical boundaries, that is to say, it has incorporated the sociocultural approach to investigate teacher agency, which has greatly enriched the knowledge base of teacher agency as well as teachers' professional development. In addition, it is clear that this study has not only attested to the applicability of the Triadic Reciprocity Framework on teacher agency (Jenkins, 2020), but also enhanced the research-based improvements by foregrounding the developmental nature of teacher agency. Therefore, as we see it, this study is very encouraging and valuable as it has provided an insightful empirical study that could support researchers to conduct more comprehensive research studies of teacher agency. Since teachers are directly involved in the school environment, this study helps school leaders and administrators recognize the importance of creating more favorable working conditions for teachers to take initiatives in teaching, rather than merely follow the policy blindly and stiffly and consequently put high pressure on teachers.

To be honest, we would also like to acknowledge some limitations of this research study if there are any. Firstly, just as the authors pointed out, the sample size of this study was relatively small and was confined to the Hong Kong context, so future studies should increase the number of samples and conduct similar research in different contexts. Secondly, teachers of different gender must also be carefully considered as the present participants were solely female teachers.

In conclusion, we find this article most informative, thought-provoking and illuminative with theoretical and practical values. Through careful reading, we believe readers will have a better and deeper understanding of the interplay among teacher beliefs, teacher agency and teachers' professional development. Hence, we would like to recommend this article without any hesitation to anyone who is interested in conducting language teacher agency from a more comprehensive and kaleidoscopic perspective.

## Author Contributions

XH drafted the General Commentary. XZ helped XH to select the commented article, provided insights and suggestions during her writing, and did the revision for the text. All authors contributed to the article and approved the submitted version.

## Conflict of Interest

The authors declare that the research was conducted in the absence of any commercial or financial relationships that could be construed as a potential conflict of interest.

## Publisher's Note

All claims expressed in this article are solely those of the authors and do not necessarily represent those of their affiliated organizations, or those of the publisher, the editors and the reviewers. Any product that may be evaluated in this article, or claim that may be made by its manufacturer, is not guaranteed or endorsed by the publisher.
